# Partial Complementarity of the Mimetic Yellow Bar Phenotype in *Heliconius* Butterflies

**DOI:** 10.1371/journal.pone.0048627

**Published:** 2012-10-31

**Authors:** Luana S. Maroja, Rebecca Alschuler, W. Owen McMillan, Chris D. Jiggins

**Affiliations:** 1 Department of Biology, Williams College, Williamstown, Massachusetts, United States of America; 2 Smithsonian Tropical Research Institute, Panama City, Panama; 3 Department of Zoology, University of Cambridge, Cambridge, United Kingdom; University of Arkansas, United States of America

## Abstract

*Heliconius* butterflies are an excellent system for understanding the genetic basis of phenotypic change. Here we document surprising diversity in the genetic control of a common phenotype. Two disjunct *H. erato* populations have each recruited the *Cr* and/or *Sd* loci that control similar yellow hindwing patterns, but the alleles involved partially complement one another indicating either multiple origins for the patterning alleles or developmental drift in genetic control of similar patterns. We show that in these *H. erato* populations *cr* and *sd* are epistatically interacting and that the parental origin of alleles can explain phenotypes of backcross individuals. In contrast, mimetic *H. melpomene* populations with identical phenotypes (*H. m. rosina* and *H. m. amaryllis*) do not show genetic complementation (F_1_s and F_2_s are phenotypically identical to parentals). Finally, we report hybrid female inviability in *H. m. melpomene* × *H. m. rosina* crosses (previously only female infertility had been reported) and presence of standing genetic variation for alternative color alleles at the *Yb* locus in true breeding *H. melpomene melpomene* populations (expressed when in a different genomic background) that could be an important source of variation for the evolution of novel phenotypes or a result of developmental drift. Although recent work has emphasized the simple genetic control of wing pattern in *Heliconius*, we show there is underlying complexity in the allelic variation and epistatic interactions between major patterning loci.

## Introduction

Recent advances in molecular genetics and genomics are leading to an in-depth understanding of the genetic basis of adaptive traits [1], [2], [3], [4], [5]. For example, warning coloration in mimetic *Heliconius* butterflies is now well understood and many genes are now mapped and characterized [5], [6], [7], [8], [9], [10]. Nonetheless, many questions remain about the genetic basis for adaptive evolution in *Heliconius*. For example, it is unclear whether similar but geographically disjunct color pattern phenotypes share a single origin or appeared independently in various populations as suggested by mtDNA analyses [11], [12]. Recent phylogeographic analysis of the *optix* gene has shown that the Amazonian rayed pattern has a single recent origin and has spread across the species range, isolating ancestral red banded forms into disjunct populations [9], [13]. However, because the genome is a mosaic, different genes should experience different evolutionary trajectories, and thus other wing patterning genes might have different evolutionary histories. Ultimately, we would like to know how commonly evolutionary convergence occurs through novel mutations, as compared to a shared evolutionary origin, and how often the same genes are implicated in controlling similar phenotypes [14].

**Figure 1:**
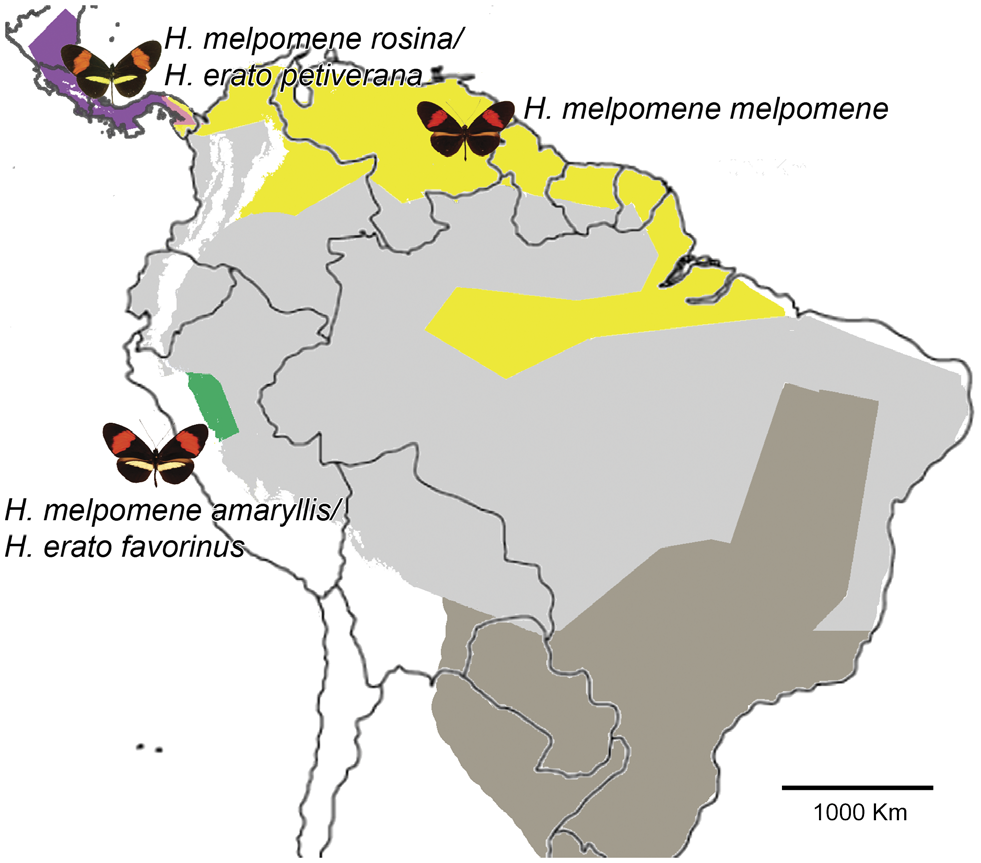
Geographical distribution of *Heliconius melpomene* and *H. erato*. Colored regions represent focus species/race distributions (each colored area has both *H. erato* and the co-mimetic *H. melpomene*), light grey areas represent areas where *H. melpomene* and *H. erato* overlap and dark grey areas represent areas where only *H. erato* occurs. Map based on [37] and [38].

**Figure 2:**
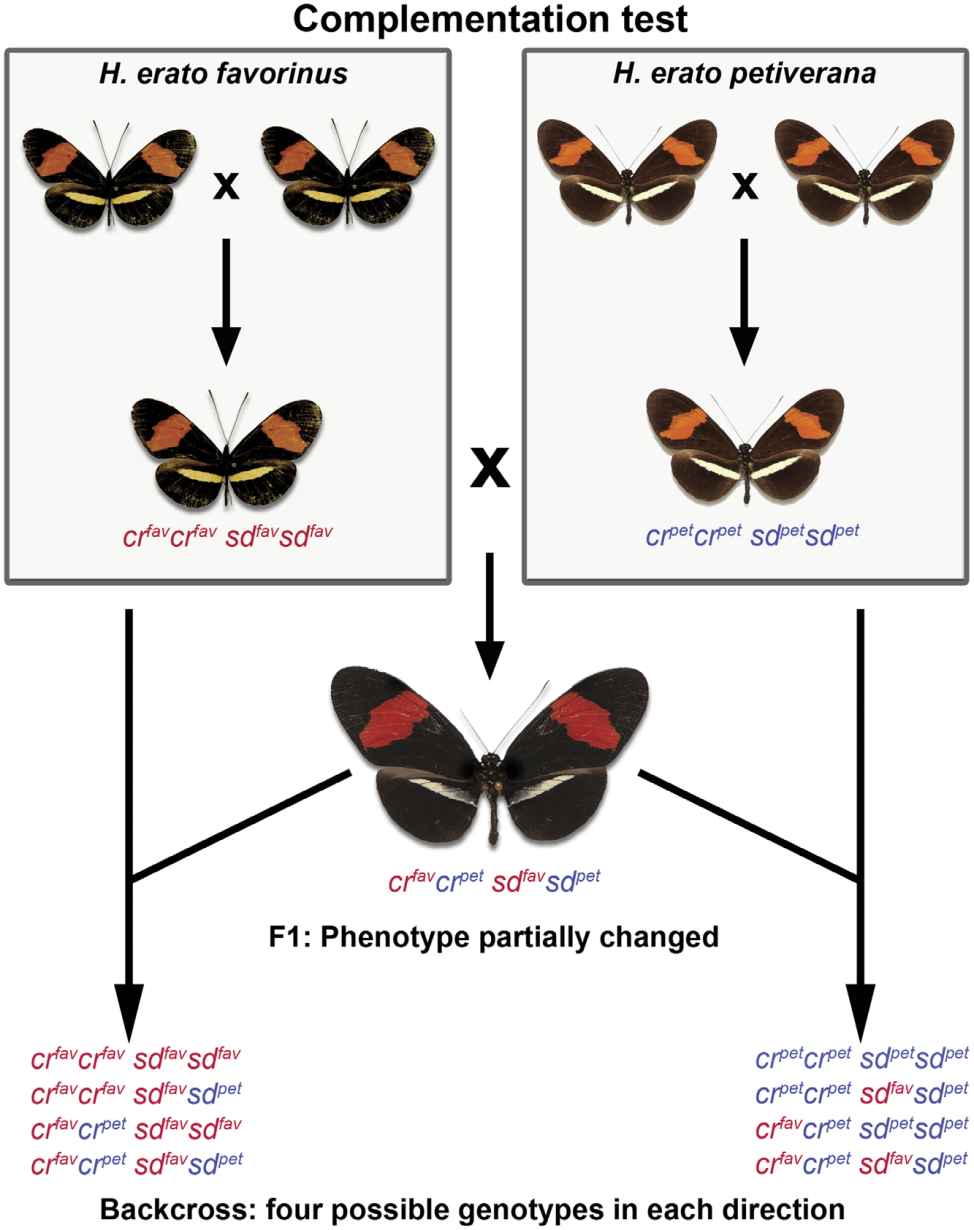
Complementation test. In a complementation test, alleles are said to complement each other if the F1 offspring exhibits the dominant phenotype (as opposed to parental recessive phenotype). The yellow hindwing bar in each race is caused by a recessive allele (*yb*) that in some cases interact with the forewing band allele (*sd*). Here both races are homozygote recessive for alleles known to be involved in color patterning (*ybyb sbsb*), however the F1 offspring exhibits a modified yellow bar, showing partial complementation. Each backcross exhibits four possible genotypes, alleles from each race are indicated by different colors and superscripts (pet = *H. e. petiverana*, fav = *H. e. favorinus*).

*Heliconius erato* and *Heliconius melpomene* are two co-mimic species that diverged 13–26 million years ago [15]. They share wing patterns wherever they coexist, but show divergent phenotypes across the neotropics [16] (Fig. 1). Here, we have used a crossing and complementation approach, combined with the use of molecular markers linked to known wing patterning loci, in order to investigate the origins of the yellow bar phenotype in these two species. Our objectives were to investigate whether allopatric races with similar wing patterns show genetic complementation (Fig. 2), a result that would indicate either independent origins of color patterning loci or divergence in allopatry. We also investigated if genetic variation for wing patterning loci exists within pure-breeding populations, a pattern that could be a consequence of divergence in allopatry or provide the necessary variation for an independent origin of patterning loci. In *H. erato*, the presence (*cr*/*cr*) or absence (*Cr*/−) of a hindwing yellow bar phenotype is controlled by the locus “Cream rectangles” (*Cr*) [17], [18], [19], [20]. In Peruvian populations the *Cr* locus interacts with the locus “Short band” (*Sd*) which affects the shape of the forewing band (*sd/sd* complete band) while in Central America the *Cr* locus alone controls the yellow band [17], [19], [20], [21]. Thus, even though the phenotypes might be similar between geographical areas, their genetic control may differ [20]. In *H. melpomene*, the presence (*yb*/*yb*) or absence (*Yb*/−) of a hindwing yellow bar is controlled by the locus “Yellow bar” (*Yb*) [18], [19], [22]. Even though the presence of a yellow bar is a recessive trait in both *H. erato* and *H. melpomene*, heterozygotes can be identified by presence of a shadow of melanic scales with altered reflectance caused by a different scale morphology [18]. The *H. melpomene Yb* locus is homologous to the *H. erato Cr* locus [6], but it is not known to interact with other loci in control of the hindwing bar [19].

We report results from five crosses, two involving *Heliconius erato* (2 races) and three involving *H. melpomene* (3 races) (Fig. 1). We show that in *H. erato*, races with nearly identical wing color patterns (Fig. 2) are under different genetic control, and indeed in one cross, the F_1_ offspring showed a pattern not observed in nature (Fig. 2). We report how some genes, previously reported to behave in a Mendelian dominant/recessive fashion [6], [7], [18], [19], [23], [24], interact to produce the observed phenotypes and that there is cryptic genetic variation in natural populations. Finally, we report that the reproductive isolation observed between *H. m. melpomene*×*H. m. rosina* is stronger than previously reported, including hybrid female inviability as well as infertility [25].

## Materials and Methods

### (a) Butterfly Collection

We used five parental races in our crosses, *H. erato petiverana, H. erato favorinus, H. melpomene rosina, H. melpomene melpomene,* and *H. melpomene amaryllis.* The pair of mimics from Panama, *H. melpomene rosina* and *H. erato petiverana,* were collected in Gamboa (9° 7′.4″N, 79° 42′.2″W) during the course of the experiment (2009–2010). Individuals of *H. melpomene melpomene* were collected near Cayenne, French Guiana (4° 54.8′N, 52° 21.6′W) in 2009. The mimics *H. melpomene amaryllis* and *H. erato favorinus* were collected in Tarapoto, Peru (6° 27′.7″S, 76° 20′.52″W) in 2008. Adults had *ad libitum* access to *Psiguria* flowers and an artificial nectar solution (10% solution of sugar and commercial bee pollen). Larvae were fed on shoots of *Passiflora biflora* (*H. erato* and *H. melpomene*) or *Passiflora menispermifolia (H. melpomene).*

### (b) Crosses and Pattern Scoring

**Figure 3:**
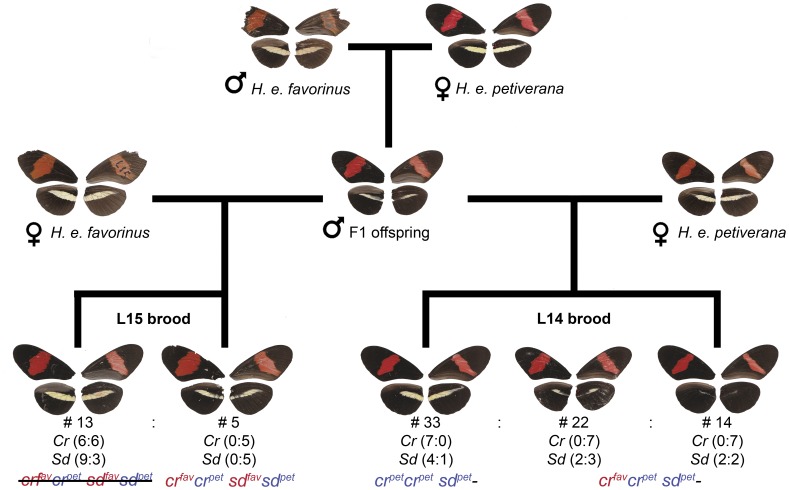
Results of crosses between *H. e. petiverana* and *H. e. favorinus*. Individuals are shown as a composite of the ventral and dorsal side of each wing (left dorsal, right ventral). For the backcrosses, F_1_ male offspring were mated to female *H. e. favorinus* (L15 brood) and *H. e. petiverana* (L14 brood). The outcome of the *H. e. petiverana* included a third wing phenotype, not present in parental or F_1_ hybrid (almost completely black hindwing). Both backcross broods had a 1∶1 sex ratio. Numbers below brood indicate number of individuals (#) with each phenotype, results of genotyping for *Cr* and *Sd* loci (parental: hybrid) and genotype (or lack of, showed as a crossed genotype) using Fig. 2 notation. In the backcross to *H. e. favorinus* (L15 brood), all individuals with an F_1_ like phenotype were hybrid (Fav/Pet alleles) for both *Cr* and *Sd* while parental like individuals were homozygotes for at least one locus. In the backcross to *H. e. petiverana* (L14 brood) all individuals with a full yellow hindwing bar were homozygotes for the *H. e. petiverana Cr* allele, however the *Sd* locus genotype did not show an association with wing color pattern.

**Figure 4:**
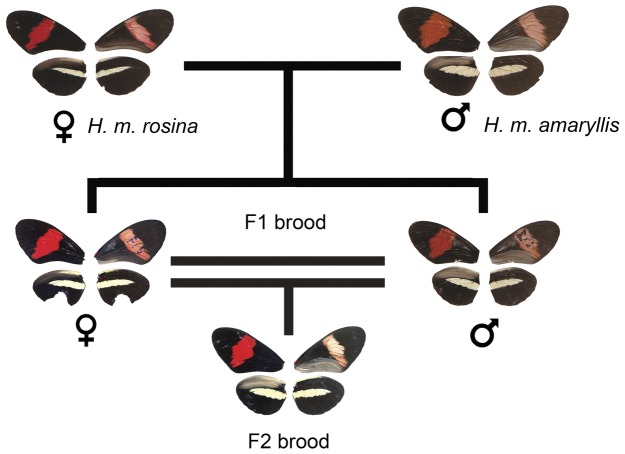
Results from crosses between *H. m. rosina* and *H. m. amaryllis*. Individuals are shown as a composite of the ventral and dorsal side of each wing (left dorsal, right ventral). Both F_1_ and F_2_ brood had a wing pattern similar to parental races (presence of a full yellow hind wing bar) and a 1∶1 sex ratio.

To test for genetic complementarity of similar wing color patterns (Fig. 2), we carried out crosses between *H. erato petiverana* and *H. erato favorinus* and between *H. melpomene rosina* and *H. melpomene amaryllis.* These represent two allopatric pairs of mimetic butterflies: *H. m. rosina* and *H. e. petiverana* are from Panama while *H. m. amaryllis* and *H. e. favorinus* are in Peru (Fig. 1). These are disjunct populations of similar phenotypes, separated geographically by the more recently derived rayed phenotypes found across the Amazon basin. All of these races share the nearly identical color pattern of forewing red band and hindwing yellow bar that differ only slightly the shape and width of the yellow and red bars (see Fig. 2, 3, and 4).

Because the F_1_ hybrids from the *H. e. petiverana* and *H. e. favorinus* cross were phenotypically distinct from their parents, we backcrossed F_1_ males to females of both parental races (L14 and L15 crosses, Fig. 3). The F1 phenotype was characterized by a broken yellow bar in which melanic scales invaded the proximal region of the yellow bar (Fig. 2) giving it a fuzzy, broken appearance as opposed to the sharp bar observed in the parental races. The F1 phenotype was particularly pronounced on the ventral side (Fig. 3). In contrast, F_1_ individuals from the *H. m. rosina* and *H. m. amaryllis* cross were not phenotypically distinct from the parental races, For this cross, we collected only F_2_ individuals (L2 cross, Fig. 4).

**Figure 5:**
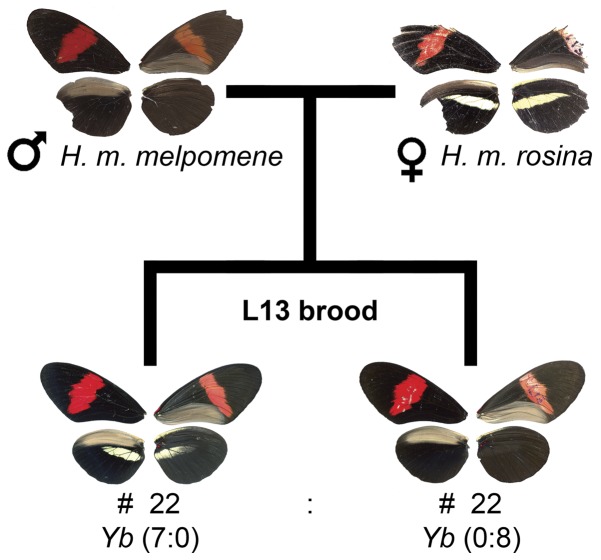
Results from crosses between *H. m. melpomene* and *H. m. rosina.* Individuals are shown as a composite of the ventral and dorsal side of each wing (left dorsal, right ventral). Unexpectedly the F_1_ brood was segregating for presence of yellow hindwing bar (22 out of 44 individuals). Half of the individuals with yellow hindwing bar had a fuzzy bar (n = 11) as show in this picture; the other half had a yellow bar similar to the maternal race (n = 11). Brood sex ratio was skewed towards males (3∶1). Numbers below phenotypes indicate number of individuals (#) with each phenotype and results of *Yb* locus genotyping (presence of paternal allele a_1_: presence of paternal allele a_2_). All individuals with yellow hindwing bar had the paternal allele a_1_ whereas all individuals with paternal allele a_2_ had a shadow hindwing bar.

We also crossed *H. melpomene melpomene* and *H. melpomene rosina* (L13 cross, see Fig. 5), a cross known for its one-way female F_1_ sterility and co-dominant yellow hindwing bar [25]. Individuals from French Guiana exhibit a completely black hindwing, while *H. melpomene rosina* have a yellow bar (Fig. 5). We were expecting to see a co-dominant pattern of inheritance, where the heterozygotes exhibit a shadow bar [6], [18]. For this cross we collected and analyzed only the F_1_ offspring.

### (c) Genetic Analysis

We genotyped offspring for markers known to be tightly linked to wing patterning genes in both species (Table 1), notably the *Yb* locus (yellow hindwing bar locus for *H. melpomene*, Genbank accession numbers FP578989, FP102339*)*, *Cr* locus (yellow hindwing bar locus, Genbank accession numbers GU583069 and CR974474), and *Sd* locus (fore and hindwing patterning gene, Genbank accession number HE668478) for *H. erato* [6], [8], [26], [27]. The *Yb*, *Cr,* and *Sd* linked locus primers have been previously used for mapping and assembly of the *Yb* and *Cr* locus regions [27], [28], [29], [30]. There were no fixed differences between races for these genotyped loci; however, there were fixed differences between individuals used in the cross. The markers used here were not necessarily the patterning genes since these remain unknown. We then calculated the probability of a random association between allele (assuming equal segregation) and phenotype (known) for each of the broods.

**Table 1:** Primer sequences for loci used in *Cr* and *Yb* loci genotyping, number of informative SNP positions* and Genbank accession numbers for the F1 sequence.

locus	Forward primer	Reverse primer	# SNPs	Accession number
*H. erato* (*Cr* locus region)				
ReqQ	TGCTACAGCTCATGTTCTGTCTG	CCCTTTTGTCTGAATGGAACTGGT	10	JX514430
LRR (Gn26)	CGTGAAGTACCGACTGTTGTAC	CATAATTTCTCAGGGAGCATACAT	9	JX514431
*H. erato* (*Sd* locus region)				
Mat	CGGGGACGTTTTAGACAGC	TGCAAAATCCTCCTCCTTTTT		JX514432 (L14) and JX514433 (L15)
*H. melpomene* (*Yb* locus region)				
B9	TGCGAGAATCTGGAGTAACAAA	GGTCTACCAGCTCTGGATGC	1	JX514429
Parn	AGTCCTCAGGCAGAGGTTGA	TGGGAAGAGTTTGAGGAAGC	1	JX514428

* Informative positions for *H. erato* were those in which *H. e. favorinus* and *H. e. petiverana* differed, for *H. melpomene* they were positions that were polymorphic in one of the parents.

All brood individuals were collected soon after adult emergence and preserved in 100% alcohol or DMSO/salt buffer. All parents and at least one grandparent were preserved soon after death and yielded high quality DNA. Genomic DNA was isolated from the thoracic region with DNeasy, Qiagen. Polymerase chain reactions (10 µl volume) contained 3 mM MgCl2, 0.2 mM dNTPs, 50 mM KCl, 20 mM Tris (pH 8.4), 2.5 ηg of each primer (see Table 1), 1 U of Perfect *Taq* DNA polymerase (5′ Prime) and 1 µl DNA. PCR amplifications were performed using a thermalcycler (Multigene, Labnet) under the following conditions: 35 cycles of 50 s at 95°C, 60 s at 48°C and 90 s at 72°C. The fragments were sequenced with an ABI PRISM 377 automated sequencer using BigDye terminator labeling (Applied Biosystems). Sequences were analyzed and alleles were scored with Lasergene seqman version 7.1.0 (DNASTAR). For the backcross to *H. e. favorinus*, we genotyped some of the offspring (n = 14 out of 17) with the restriction enzyme *AseI* (Thermo Scientific, Fermentas), that has a recognition sequence that includes a diagnostic SNP site (Y). We fully digested 5 µl of PCR product (*Sb* linked Mat) with *AseI* and ran the digests on a 2% agarose gel using the F1 father, the *H. e. favorinus* mother, and previously genotyped offspring as controls. We could unambiguously assign all offspring as homozygotes or heterozygotes for the diagnostic SNP.

### Ethics Statement

We obtained all necessary permits for the described field collections. For butterflies collected in Panama, we obtained ANAN permit (SE/A-28-10). For butterflies collected in Peru, we obtained permits from the Peruvian Ministerio de Agricultura and Instituto Nacional de Recursos Naturales (004-2008-INRENA-IFFS-DCB and 011756-AGINRENA). French Guiana is part of the EU and does not require collection permits for research. None of the collection locations were privately-owned and *Heliconius* butterflies are not endangered or protected.

## Results

### The *H. erato* petiverana and *H. erato* favorinus cross

Crosses between these two *H. erato* races exhibited a very similar phenotype (forewing red band and hindwing yellow bar) and produced F_1_ hybrids in which the yellow bar was fuzzy and broken (Fig. 2, 3 and methods). This demonstrates that genetic control of this phenotype is divergent between the two populations, consistent with previous data showing the yellow band of *H. e. favorinus* is controlled by both *Cr* and *Sd* loci, while that of *H. e. petiverana* is controlled by the *Cr* locus alone [20]. To investigate the genetic basis for the breakdown of this phenotype in the F_1_, we conducted backcrosses in both directions.

#### Backcross to *H. e. petiverana*

For the backcross to *H. e. petiverana,* we reared 69 individuals; 33 were parental-like (yellow hindwing bar), 22 were F_1_ like (fuzzy bar) and 14 had black hindwings with very few yellow scales. The latter phenotype was never observed in either parental or F_1_ generations and was characterized by the almost complete absence of yellow scales (Fig. 3): these phenotypes represented three distinct classes (Fig. 3). If presence of a full yellow bar is recessive, then we would expect a 1 (yellow bar):1 (F_1_ like + black) ratio, as was observed (χ^2^ = 0.03, df = 1, p = 0.72).

Previous crosses [6] have indicated that the yellow bar in *H. erato* is primarily controlled by the *Cr* locus. To test the involvement of this locus here, we genotyped loci located in the mapped color region (*Cr* locus) (Table 1 and 2). In the backcross to *H. e. petiverana*, all tested individuals with a full yellow hindwing bar (n = 7) carried only *H. e. petiverana* alleles, whereas all individuals that were phenotypically F_1_ like (n = 7) or had black hindwings (n = 7) were hybrids, here defined as one allele of each race. The chance of a random association between *Cr* allele and color for the 21 individuals analyzed was only 4.7×10^−7^. For the *Sd* locus (Table 1 and 2) there was no association of allelic origin with wing color pattern. Individuals with *H. e. petiverana* alleles only, as well as hybrid individuals, were found for all color patterns. Contrary to expectations, this result indicated that the partial genetic complementation between *H. e. petiverana* and *H. e. favorinus* was not only due to the additional involvement of the *Sd* locus in the Peruvian race, but also due to divergent *Cr* alleles in the two races. Although the F1 like and black hindwing individuals could not be differentiated genotypically, it was possible that the latter phenotype was caused by a yet unidentified third locus that interacts epistatically with the *Cr* locus.

**Table 2:** Diagnostic single nucleotide polymorphism (SNP) positions (based on Genbank sequence, see Table 1) for each locus.

*H. erato Cr* region					
Locus: *LRR*			Locus: *ReqQ*		
position	*H.e.pet*	*H. e. fav*	position	*H.e.pet*	*H. e. fav*
49	A	G	55	T	C
154	A	T	302	T	C
195	C	A	352	G	A
267	A	T	362	A	G
330	A	T	365	A	T
493	C	A	366	A	C
552	T	C	376	C	T
572	C	G	391	G	A
581	A	C	464	A	T
–	–	–	469	C	T
***H. erato Sb*** ** region (locus: ** ***Mat*** **)**					
backcross to *H. e. favorinus*			backcross to *H. e. petiverana*		
position	F1	Mother (*fav*)	position	F1	Mother (*pet*)
85	R (G from *pet*)	A	281*	M (C from *fav*)	C
301*	Y (C from *pet*)	C	–	–	–
327*	Y (C from *pet*)	C	–	–	–
358	W (T from *pet*)	A	–	–	–
430	Y (T from *pet*)	C			
***H. melpomene Yb*** ** region**					
Locus: *B9*			Locus: *PARN*		
position	Father (*mel*)	Mother (*ros*)	position	Father (*mel*)	Mother (*ros*)
258	R	T	186	Y	C

Abbreviations: pet = *H. e. petiverana;* fav = *H.e. favorinus;* mel = *H.m.melpomene;* ros = *H.m. rosina.*

For the *Cr* locus region, all heterozygotes had a hybrid genotype (i.e. one allele from *H.e.favorinus* and one allele from *H.e.petiverana*).

* At the *Sb* region, some of the diagnostic SNPs were polymorphic, as a result, the hybrid genotype (i.e. one allele from *H.e.petiverana* and one allele from *H.e. favorinus*) was actually homozygote.

#### Backcross to *H.e. favorinus*

In the backcross to *H. e. favorinus* there were only two phenotypes, F_1_ like and parental-like. We reared 18 individuals for the backcross to *H. e. favorinus*, and of these, 13 were parental like (yellow bar) and five were F_1_ like (shadow bar), which deviated from the expected 1∶1 segregation if only one gene was controlling color pattern (χ^2^ = 3.56, df = 1, p = 0.06). Unlike the backcross to *H. e. petiverana,* the *Cr* locus did not explain color pattern variation. Although all F_1_ like individuals were *Cr* hybrids, i.e. one allele from each race as expected, the yellow bar individuals included both hybrids and pure *H. e. favorinus* individuals (Fig. 1). For the *Sd* locus. all F_1_ like individuals were again hybrids while the yellow bar backcross individuals included both hybrids and pure *H. e. favorinus* alleles. However, no yellow bar individual was a hybrid at both *Cr* and *Sd* simultaneously. In summary, the most parsimonious hypothesis was that a hybrid genotype at both *Cr* and *Sd* loci is necessary for the expression of the F_1_ like ‘shadow bar’ phenotype. This should give a 1∶3 ratio consistent with that observed from our data (χ^2^ = 0.074, df = 1, p = 0.78). This cross is therefore similar to previous crosses, in that *H. favorinus* alleles at both *Sd* and *Cr* loci are required for full expression of the yellow bar [20], although it differs in detail in that a full homozygote genotype at both loci was not necessary for full expression of the phenotype.

### The *H. melpomene rosina* and *H. melpomene amaryllis* cross

In contrast to the mimetic races of *H. erato*, the *H. melpomene* cross between races with similar wing phenotypes produced F_1_ hybrids with parental-like wing patterns (Fig. 4). Similarly, in the F_2_ cross (n = 30) there was also no yellow band wing phenotype variation. In this cross we did not observe genetic complementation suggesting that the alleles found in the different races act in a similar way to produce the yellow hindwing bar.

### The *H. melpomene melpomene* and *H. melpomene rosina* cross

When a male *H. m. melpomene* is crossed with a female *H. m. rosina*, the female offspring are sterile, while in the reverse direction both sexes are fertile [25]. Furthermore, heterozygotes at the *Yb* locus exhibit a visible shadow bar with different scale morphology, but no yellow pigment [18]. In addition to investigating wing pattern variation, we also aimed to confirm the inheritance of sterility in this cross. In most cases hybrid offspring were identical and showed a shadow band as expected. However, in a single cross, individuals with both a shadow bar (n = 22) and fuzzy pigmented yellow bar (n = 22) were present in the F_1_ generation.

Genetic analysis showed that there was a perfect association between inheritance of one of the paternal (*H. m. melpomene*) alleles at the *Yb* locus (thus an allele for lack of hindwing bar) and offspring phenotype (Fig. 5). The chances of a random association between one paternal allele and wing phenotype for the 15 individuals analyzed is 3×10^−5^. This indicated that there is standing genetic variation at the *Yb* locus within *H. m. melpomene* that influences expression of the yellow bar.

Consistent with previous findings [25], all five females tested were completely sterile. Although they laid eggs at a normal rate (2–5 eggs a day), none of the eggs hatched. Unexpectedly, the brood showed a deviation from the expected 1∶1 sex ratio where of 44 individuals, only 10 were females (χ^2^ = 13, df = 1, p<0.001). These results suggest that females were not only sterile but also partially inviable, possibly due to larval mortality as high as 90%, as egg hatching rate was within normal range, above 90%.

## Discussion

Our results demonstrate surprising diversity in the genetic control of a common phenotype in both *H. erato* and *H. melpomene*. In *H. erato*, populations in Panama and Peru with nearly identical phenotypes were already known to differ in different genetic of yellow bar. Two loci, *Cr* and *Sd*, control a phenotype in Peru that is controlled by only the *Cr* locus in Panama [17], [19], [20], [21]. However, we have further shown that alleles at the *Cr* locus in the two populations show partial genetic complementation. i.e. F_1_ hybrids did not resemble the parental phenotype, although the pattern did not entirely disappear, perhaps suggesting independent origins for similar patterns in these disjunct populations. Additionally, we have shown that there was cryptic diversity segregating within the Panamanian *H. melpomene* population at the *Yb* locus that could be an important source of variation in the evolution of novel phenotypes.

Thus, although all populations of *H. erato* recruited *Cr* and/or *Sd* loci as their yellow hindwing patterning genes, the alleles in each population are not the same and partially complement one another. This situation is similar to parallel evolution in freshwater sticklebacks, *Gasterosteus aculeatus*, where the loss of pelvic spines occurred independently multiple times [31], and to the parallel evolution of cave-dwelling fish, *Astyanax mexicanus* [1], where striking phenotypic convergence occurred independently at least five times [32]. In sticklebacks, the same *Pitx1* locus was recruited [4], [33], [34] for pelvic spine reduction; however, the partial complementation observed between populations of similar phenotype, and sequencing of the *Pitx1* locus indicates that different alleles are involved in each population [4], [31], [34]. Similarly, in *A. mexicanus* the same gene is implicated in albinism, through similar, but independent, deletions [1].

In the case of *Heliconius*, the evolution of multiple alleles controlling similar phenotypes can be explained by two phenomena, either multiple origins for patterning alleles in disjunct locations, or alternatively a common origin followed by divergence in the genetic control of a shared phenotype, a phenomena that has been termed ‘developmental drift’ [35]. Analysis of the *Heliconius* genome showed that introgression of color pattern genes between species played a role in the evolution of mimicry [10], whereas mtDNA and AFLP data suggested independent origins of color patterns [11], [12]. These results might suggest that color patterning genes can also spread through populations of the same species, perhaps at a faster rate than mtDNA or other non-color linked genes. However, a final answer to this question will require a phylogeographic analysis similar to that already carried out for the red patterning gene *optix* [13]. That is, if *Cr* and *Sd* loci lineages cluster by color pattern instead of by geography, this would support a single origin followed by diversification.

As reported previously [20], the epistatic interaction between *Cr* and *Sd* involved in yellow hindwing color patterning of *H. erato* was observed in only one of the backcrosses. In the backcross to *H. e. petiverana,* the presence of the yellow bar is explained by *Cr* locus alone, whereas in the backcross to *H. e. favorinus,* both *Cr* and *Sd* loci are needed to explain the yellow bar, i.e.a fav/fav genotype in at least one of the loci suffices for the expression of yellow hindwing bar (Fig. 1). These results corroborate observations from previous crosses indicating that in *H. e. favorinus,* both *Cr* and *Sd* loci are involved in the phenotype whereas in *H. e. petiverana, Cr* alone can explain the observed phenotype [20], [23].

In contrast to the *H. erato* results, the *Yb* alleles in the mimetic races of *H. melpomene*, i.e. *H. m. rosina* mimetic to *H. e. petiverana* and *H.m. amaryllis* mimetic to *H. e. favorinus*, failed to complement. All the F_1_ and F_2_ hybrid offspring exhibited the parental phenotype, presence of hindwing yellow bar (Fig. 4), indicating similar genetic control of the phenotype. If these different genetic controls are a result of a common origin followed by divergence in allopatry, then this result observed in *H. melpomene* might be expected. *Heliconius melpomene* is thought to be the mimic to *H. erato* [36], as *H. melpomene* has lower genetic diversity, smaller population size and a history of recent population expansion [12], [36]. Thus, the alleles controlling color variation in *H. melpomene* might be more recent, less genetically diverse and thus have had less opportunity and time to diverge between populations. This result is however surprising given the recent sequencing of *H. melpomene Yb*-linked alleles (Nadeau personal communication) which indicates that they might have different origins in the two populations or might be derived from a more common, ancient allele.

The potential for developmental drift [35] in allelic control of *Heliconius* patterns was supported by the observation of segregating genetic variation in the French Guiana *H. melpomene* population. In *H. melpomene,* yellow bar is controlled by a recessive allele at the *Yb* locus [6], [18]. Although *H. m. melpomene* individuals exhibiting a yellow bar or a shadow heterozygote bar have never been observed in French Guiana, our results indicate that standing genetic variation for presence of yellow bar exists in this *H. m. melpomene* population, and that this phenotype can be expressed when in the appropriate genetic background. This indicates, contrary to the general assumption of pure monomorphism of wing patterns in mimetic *Heliconius* populations, that there may be cryptic standing variation that could predispose populations to the evolution of novel phenotypes, either through independent origins or due to developmental drift.

Finally, we found hybrid female inviability in the *H. m. melpomene*×*H. m. rosina* cross, where previously only female sterility had been reported [25]. Our cross exhibited a biased adult sex ratio substantially skewed towards males (3∶1). We also observed very high larval mortality but normal egg hatchability, suggesting that females were less likely to survive in the larval phase. This indicates further incompatibility between these populations than has previously been recognized and represents another example of incipient reproductive isolation within a species.

In summary, it is clear that standing genetic variation, both in the genetic control of common phenotypes and as cryptic genetic variation, is more common than has often been recognized in *Heliconius* butterflies. This high level of genetic variation might contribute to divergence between populations and eventually incompatibility, such as observed here between the parapatric races of *H. melpomene*.
